# Euthanasia of Dogs by Australian Veterinarians: A Survey of Current Practices

**DOI:** 10.3390/vetsci10050317

**Published:** 2023-04-27

**Authors:** Brianne Marlene Pepper, Hedia Chan, Michael P. Ward, Anne Quain

**Affiliations:** Sydney School of Veterinary Science, University of Sydney, Camperdown, NSW 2006, Australiamichael.ward@sydney.edu.au (M.P.W.)

**Keywords:** euthanasia, veterinarian, companion animal, canine, dog, end-of-life, premedication, sedation, pentobarbitone, animal welfare

## Abstract

**Simple Summary:**

Veterinarians are commonly required to euthanise dogs in the course of their work. The way euthanasia is performed can impact the welfare of dogs, the wellbeing of the client, and the wellbeing of the veterinary team members involved. There are published guidelines regarding humane euthanasia techniques, but there are few reports on how veterinarians actually perform euthanasia, in both non-emergency and emergency contexts. We surveyed Australian veterinarians on the techniques they used, including whether they used premedication or sedation prior to euthanasia of dogs. We found that almost all veterinarians used barbiturates to euthanise dogs. The majority provided some form of premedication or sedation prior to euthanasia in non-emergency contexts, compared with just under half in emergency situations. The type of premedication or sedation varied. Factors associated with administering a premedication or sedation included the gender of the veterinarian, their location and the type of practice they worked in. Veterinarians had differing practices and views about the way in which canine euthanasia should be performed. These findings will be useful to allow individual veterinarians to benchmark and improve their own euthanasia practices and may assist in the development and refinement of canine euthanasia protocols.

**Abstract:**

Euthanasia techniques utilised by veterinarians impact the welfare of many dogs in their final moments. Despite euthanasia guidelines, little is known about euthanasia techniques used in practice. We administered an online survey of Australian veterinarians who had euthanised at least one dog in the previous 12 months. We found that 668 (96.8%) had euthanised a dog in the previous 12 months, almost all using intravenous pentobarbitone sodium (n = 651, 99.7%). For non-emergency euthanasia (n = 653), the majority (n = 442, 67.7%) administered a premedication or sedation prior to euthanasia versus less than half for emergency euthanasia (n = 286, 46.4%). Practices and views about euthanasia varied. Female veterinarians and veterinarians located in metropolitan regions were more likely to administer a premedication or sedation prior to non-emergency euthanasia (*p* < 0.05). Veterinarians in private mixed animal practices were less likely to administer a premedication or sedation prior to a non-emergency euthanasia (*p* < 0.05). For non-emergency and emergency euthanasia, veterinarians who worked in “other” practice types were more likely to administer a premedication or sedation than private companion animal practices (*p* < 0.05). The possible reasons for differences in euthanasia practices are explored, and scope for refinement is identified.

## 1. Introduction

In performing euthanasia, veterinarians seek to induce a “good death”—one that minimises the suffering, discomfort and distress of animals, with emphasis placed on effecting a painless, rapid and smooth loss of consciousness prior to death [1,2]. Indications for euthanasia or humane killing include preventing further deterioration of quality of life, relieving suffering or protecting the health and safety of animals and humans [1,3,4,5]. Caregiver burden, financial and practical constraints may also influence the decision of whether and when to euthanise a dog [6,7].

Veterinarians are commonly required to perform humane euthanasia of dogs in companion animal clinical practice [3]. In some Western countries, the majority of canine patients registered to veterinary practices die by euthanasia. For example, 87–89% of companion dogs in the United Kingdom and 91% of dogs in New Zealand registered to private veterinary practices were euthanised by veterinarians [6,8,9]. Additionally, 80% of kennel club registered dogs in the UK were euthanised [10]. Reported dog euthanasia rates were lower in Italy (41%), Brazil (48%) and Taiwan (22%) [11,12,13].

Euthanasia is considered a Day One Competency, meaning that veterinary graduates are required to recognise when euthanasia is necessary and perform it in a manner that is humane [14,15]. Competency when performing euthanasia is not only critical for the welfare of the patient but crucial for the wellbeing of veterinary clients, veterinary team members and the veterinarian–client relationship [16]. Matte and colleagues reported that the way in which veterinarians manage companion animal euthanasia correlates with either alleviating or aggravating a client’s grief [17].

Although euthanasia is expected to be a core competency of veterinarians, there is evidence that the teaching of end-of-life decision-making and euthanasia techniques is variable. For example, Littlewood and colleagues found wide variation in the teaching of end-of-life decision making and euthanasia within Australasian veterinary schools [16,18]. Not all veterinary students were exposed in assisting or performing euthanasia during their studies [18]. A study in the United States found that students were minimally exposed to euthanasia practices, with an average of just 2.8 h devoted to euthanasia methods and techniques within the veterinary curriculum [19]. A recent survey of New Zealand veterinarians (n = 361) found that one third had no formal training of key aspects of euthanasia in veterinary school, 84% had not performed euthanasia of a dog, and 13% had not observed the euthanasia of a dog during their veterinary training [20].

Numerous euthanasia methods are described for companion animals, with the intravenous (IV) injection of a barbiturate acid derivative being the method preferred by the American Veterinary Medical Association (AVMA) [1]. According to these guidelines, when IV access cannot be established in a patient, other methods—such as the intraperitoneal (IP) administration of sodium pentobarbital, non-barbiturate anaesthetic overdose and inhalation anaesthetic overdose—are also acceptable [1].

The quality of euthanasia does not exclusively depend on the method; it is also influenced by the circumstances in which euthanasia occurs, the indication for euthanasia, the environment and the method of patient restraint [21].

Until recently, the use of premedication or sedation prior to euthanasia has been described as a matter of clinician preference [3]. Current AVMA guidelines recommend that, whenever practicable, pre-euthanasia sedation or anaesthesia should be provided for canine patients [1]. The administration of a premedication or sedation prior to the euthanasia agent may reduce the incidence of adverse reactions in the patient (such as agonal gasping and muscle tremors), reduce patient stress associated with handling and relieve patient anxiety, distress and pain during the euthanasia procedure [1,21,22]. This may also decrease the risk of injuries to veterinary team members and clients from animals during euthanasia. Additionally, premedication or sedation of the patient may reduce anxiety and distress of the veterinarian and client [22]. Agents used for this purpose include benzodiazepines (e.g., diazepam, midazolam), opioids (e.g., butorphanol, methadone), alpha-2 agonists (e.g., dexmedetomidine, xylazine), phenothiazines (e.g., acepromazine), dissociative anaesthetic agents (e.g., ketamine), NDMA antagonist-benzodiazepine tranquilizer combinations (e.g., tiletamine-zolazepam), hypnotics (e.g., propofol), neurosteroids (e.g., alfaxalone) and inhalational anaesthetic agents (e.g., isofluorane) [23].

In recent years, there has been increased awareness and attempts to mitigate the potential iatrogenic harms of veterinary care in companion animal practice in general [24,25]. This extends to patients’ final moments, where there is an increasingly recognised need to reduce patient fear, anxiety and distress both before and during euthanasia [5].

Although euthanasia is a common procedure, there is scant published literature on how veterinarians in practice euthanise companion animals. Understanding current practices allows individuals to benchmark their own practices and allows researchers to identify potential areas of refinement to promote the welfare of canine patients and the wellbeing of their owners and veterinary team members. The objective of this study was to determine the methods and practices utilised by Australian veterinarians when performing non-emergency and emergency euthanasia in canine patients. A particular focus of this research was to explore the frequency of and potential predictors for administration of a premedication or sedation prior to euthanasia.

## 2. Materials and Methods

Survey questions were developed based on literature reviews and discussions with veterinarians. An online survey was built and administered by Research Electronic Data Capture (REDCap), a University of Sydney hosted, secure server-based application. The survey was piloted with a veterinary pharmacologist and three companion animal veterinarians who graduated in different decades (1990s, 2000s, 2010s). The feedback received was utilised to improve the quality of the data and incorporated into the final study version.

The survey was divided into three sections: (1) euthanasia practices in dogs, (2) euthanasia practices in cats and (3) participant demographics (see Table S1). Section 2 was further divided into questions relating to euthanasia practices associated with non-emergency euthanasia and those relating to emergency euthanasia. What constituted a non-emergency as opposed to an emergency euthanasia could be determined by respondents. This paper focuses on the subset of dog data. The questionnaire was designed using closed, categorical questions with pre-defined answers. All questions had a drop-down menu; when “other” was selected, respondents could provide an alternative response. At the conclusion of sections (1) and (2), an optional open question “Is there anything else you wish to add about your approach to euthanasia?” was included so that respondents could add any additional information.

### 2.1. Recruitment, Consent and Ethics Approval

The survey was open to all veterinarians registered in an Australian State or Territory over the age of 18 years. The survey was distributed by the Australian Veterinary Association (AVA), the NSW Veterinary Practitioners Board (NSWVPB), the Centre for Veterinary Education (CVE) and the Australian Veterinarian Network Facebook page by the publication of the REDCap survey link. The survey was open from February to June 2022. No incentives were offered to participants or distributors of the survey link.

Informed consent was gained by inclusion of a participant information statement as the survey landing page and informing the respondents that clicking the “submit” button indicated consent for their responses to be included in the study. Completion of the survey was voluntary, and respondents could exit at any point prior to submitting their responses. The responses were anonymous. At the end of the survey the participants were able to provide their email address to receive a summary of the results. This was via a link to a second survey so that email addresses could not be associated or stored with survey responses to ensure anonymity.

At the conclusion of the survey and prior to submission, participants were directed to mental health resources (The Australian Veterinary Association’s telephone counselling service, The Doctor’s Health and Advisory Service telephone helpline and Beyond Blue) in case recollection of euthanasia experiences caused discomfort or distress.

The study was approved by the University of Sydney Human Research Ethics Committee (2021/964).

### 2.2. Data Cleaning

The completed survey responses in REDCap were downloaded into Microsoft^®^ Excel^®^. Respondents that had selected “other” and stated a response that was already included in the options were recategorized into the appropriate category. Respondents who had selected “other” and whose responses were not reflected in the drop-down menu were retained. The data were reviewed for valid values before importing into IBM SPSS^®^ Statistics Version 28 (release 28.0.0.0) for statistical analysis.

### 2.3. Outcome and Explanatory Variables

The variable “years since graduation” was calculated by subtracting the graduation year from 2022.

A total of four explanatory variables were considered for regression analysis: gender, type of workplace, location and years since graduation. All variables were categorical, except for one continuous variable: years since graduation.

To facilitate statistical analysis, some categories were removed or recoded into new categories. The “other” category for gender was removed due to the low number of responses. For the explanatory variable of primary workplace, “animal shelter practice/charity/NGO”, “research laboratory” and “veterinary teaching hospital” were recoded into the “other” category. For the explanatory variable of location, “rural” and “remote” were combined and recoded to “rural and remote”.

### 2.4. Descriptive Analysis

Descriptive analyses were performed for the demographic data. The distribution of categorical variables relating to euthanasia practices in both an emergency and non-emergency scenario were assessed with frequency tables. The distribution of the continuous variable “years since graduation” was described using summary statistics (median and interquartile range).

### 2.5. Univariable Analysis

Univariable binary logistic regression analyses were performed to assess the association between the explanatory variables and the outcome variable, administration of a premedication or sedation prior to euthanasia. The assumption of linearity for the continuous variable years since graduation was checked by categorising the variable by quartiles, running a univariable model and plotting the resulting odds ratios against the midpoints of the quartile-based groups. The values were considered statistically significant if the *p*-value was <0.05. The results were reported as odds ratios (OR) and 95% confidence intervals (CI).

### 2.6. Multivariable Analysis

A multivariable binary logistic regression model was built using a backward elimination approach. The variables were considered statistically significant in the model if the *p*-value was <0.05. The results were interpreted as OR and 95% CI. The potential confounding by “years since graduation” was assessed by calculating the percent change in the regression parameters of variables in the final model when the potential confounder was added to the final model. An estimated change of more than 20% was considered to indicate substantial confounding, warranting inclusion of the variable in the final model, irrespective of its *p*-value. Model fit was assessed by comparing the fitted model to the intercept-only model.

### 2.7. Coding of Free-Text Responses

The free-text responses were analysed according to the principles of thematic analysis (TA) using an inductive approach aligned with codebook TA [26,27]. The free-text responses were read repeatedly by three authors (BP, HC and AQ) to ensure familiarity with the data. A codebook was developed based on a review of these responses and the literature on companion animal euthanasia. The responses were transferred into an MS Word document and uploaded onto NVivo (Release 1.7.1 (1534)) (QSR International) to facilitate coding. Extracts of the free-text response from a single respondent could be coded multiple times if the comment crossed multiple codes.

The codes were reviewed for internal coherence and distinctiveness from other codes, which involved re-reading all coded extracts of comments for each code. Where extracts did not fit a code, they were recoded. Finally, a table was constructed to depict the frequency of codes, with examples of coded extracts. This was implemented to indicate the prominence of codes relative to one another. Although this is not typical of a TA approach [28], this approach has been used in previous veterinary studies involving large numbers of free-text responses comprising a large breadth but shallow depth of data [29].

## 3. Results

A total of 705 respondents commenced this survey, of which 690 responded to the question as to whether they had euthanised a dog in the previous 12 months. When respondents were asked if they had euthanised a dog in the previous 12 months, 668/690 (96.8%) of the respondents answered yes and 22/690 (3.2%) answered no; those responding no were subsequently excluded from the analysis.

Of the remaining 668 responses, 598 participants completed the demographics section of the survey for the subset of data relating to dogs. The distribution of categorical demographic variables for these 598 respondents is described in Table 1. Briefly, most respondents were female (n = 490, 81.5%) and worked in a private companion animal practice (n = 433, 72.4%). Those who selected “other” listed their caseload comprising mobile veterinarian, consultancy, university academic, emergency and referral clinic, corporate companion animal clinic, government veterinarian, animal welfare veterinarian, wildlife veterinarian or primarily single species veterinarian (“goat only”, “equine only”, respectively). The majority of respondents either worked in a metropolitan (n = 287, 48.0%) or regional (n = 263, 44.0%) location, with a smaller proportion of veterinarians working in rural and remote locations (n = 48, 7.2%). Respondents graduated between 1 and 55 years ago, with a median of 11 years and interquartile range of 4–23 years.

### 3.1. The Administration of a Premedication or Sedation during Euthanasia

The majority of respondents (n = 442/653, 67.7%) administered a premedication or sedation in a non-emergency euthanasia. In contrast, a smaller percentage of respondents administered a premedication or sedation (n = 286/616, 46.4%) for emergency euthanasia. When respondents were asked the reasoning behind the use of a premedication or sedation, the most frequently selected reasons were to reduce stress to the patient (non-emergency: n = 410, 92.8%; emergency: n = 259, 90.6%), reduce stress to the owner (non-emergency: n = 363, 82.1%; emergency: n = 184, 64.3%) and as a form of chemical restraint (non-emergency: n = 162, 36.7%, emergency: n = 114, 39.9%). Only a small percentage of respondents were taught to administer a premedication or sedation prior to the euthanasia agent for both non-emergency and emergency euthanasia (non-emergency: n = 41, 9.3%; emergency: n = 22, 7.7%). Of the responses that selected “other”, the most frequent reasons for administration of a premedication or sedation were to reduce the likelihood of adverse reactions (e.g., agonal gasping, muscle fasciculations and vocalisation) from the euthanasia agent and to reduce stress to the veterinarian (see Table S2 for all other reasons).

The most common drugs administered in a non-emergency euthanasia were tiletamine-zolazepam (n = 200, 45.2%), acepromazine (n = 170, 38.5%) and opioids (n = 154, 34.8%) (for all drugs listed, see Table S2). In comparison, the most common drugs administered in emergency euthanasia were opioids (n = 147, 51.4%), tiletamine-zolazepam (n = 94, 31.8%) and acepromazine (n = 65, 22.7%) (for all drugs listed, see Table S2).

When asked about the route of administration of the premedication or sedative drugs, most respondents selected IV injection (n = 165, 37.3%), subcutaneous injection (n = 159, 36.0%) and intramuscular injection (n = 154, 34.8%) in a non-emergency euthanasia (for all drugs listed, see Table S2). For an emergency euthanasia, a higher proportion of respondents selected IV injection as their primary route of administration of premedication or sedation administration (n = 146, 51.0%), followed by intramuscular injection (n = 96, 33.6%) and subcutaneous injection (n = 52, 18.2%) (for all drugs listed, see Table S2). Pentobarbitone sodium was the primary euthanasia agent administered, with 99.7% (n = 651) of respondents using this agent during a non-emergency euthanasia and administering it intravenously (n = 649, 99.5%) (for all options listed, see Table S2).

### 3.2. Descriptive Results for Practices Relating to Euthanasia

Non-emergency and emergency euthanasia practices are described in Table 2. Most canine euthanasia was performed in the veterinary clinical setting (n = 552, 84.7% of non-emergency euthanasia and 89.9% (n = 257) of emergency euthanasia, respectively). A smaller proportion (n = 97, 14.9% of non-emergency euthanasia and n = 27, 9.4% of emergency euthanasia) were performed as a house-call or home visit. The owner was present in 95.4% (n = 622) of non-emergency euthanasia and 89.9% (n = 257) of emergency euthanasia. Most veterinarians scheduled 30 (n = 386, 59.2%) or 20 (n = 116, 17.8%) min for non-emergency euthanasia appointments. Euthanasia appointment lengths ranged from 10 min to unlimited. For both non-emergency and emergency euthanasia, veterinarians were usually assisted (non-emergency: n = 449, 68.9%; emergency: n = 197, 68.9%), most frequently by a veterinary nurse (non-emergency: n = 436, 97.3%; emergency: n = 192, 97.5%), though occasionally by a client (non-emergency: n = 8, 1.8%; emergency: n = 3, 1.5%) (for all options listed, see Tables S2 and S3). When asked about the adjunctive measures associated with the euthanasia, “away from other animals”, “soft bedding” and “longer appointment time” were employed the most in both non-emergency and emergency euthanasia. Treats were administered more commonly in non-emergency than emergency euthanasia, while an absence of adjunctive measures was more common in emergency euthanasia. For non-emergency euthanasia, the majority of participants (n = 607, 93.1%) did not dispense medication for the patient prior to the appointment. Where pre-visit pharmaceuticals were dispensed, the most common were gabapentin (n = 29, 64.4%) and trazadone (n = 26, 57.8%) (see Table S2 for all options listed).

### 3.3. Predictors for the Administration of a Premedication or Sedation during Euthanasia

The assumption of linearity for the continuous variable years since graduation was not met for either of the two outcomes, hence, the variable was included as a categorical variable in modelling. Predictors for the administration of a premedication or sedation during a non-emergency euthanasia are described in Table 3 and Table 4. Gender, workplace, location and years since graduation were assessed in a univariable logistic regression. Female respondents were 1.9 times more likely to administer a premedication or sedation compared to males (95% CI 1.2–2.9, *p* = 0.004). The type of workplace was also significantly associated with the administration of a premedication or sedation (*p* < 0.001). Veterinarians who worked in “other” workplace practice types were 3.4 times more likely (95% CI 1.5–7.6) and veterinarians who worked in a private mixed practice were half as likely (95% CI 0.3–0.8) to administer a premedication or sedation compared to those that worked in private companion animal practices. The location of the veterinarian was also significantly associated with the administration of a premedication or sedation (*p* < 0.001). Veterinarians who worked in metropolitan regions were 3.3 times more likely (95% CI 1.7–6.1) to administer a premedication or sedation compared to veterinarians who worked in rural and remote regions. Years since graduation were not significantly associated with the administration of a premedication or sedation in a non-emergency euthanasia (*p* = 0.641).

In the multivariable analysis, gender (*p* = 0.004), location (*p* = 0.003) and workplace (*p* = 0.018) were all associated with the administration of a premedication or sedation in a non-emergency euthanasia. In this model, female veterinarians (OR 1.9, 95% CI 1.2–3.0), veterinarians in other workplaces (OR 3.5, 95% CI 1.5–7.9) and veterinarians in metropolitan regions (OR 2.5, 95% CI 1.2–5.1) were more likely to administer premedication or sedation in a non-emergency euthanasia compared to male veterinarians, veterinarians working in private companion animal practices, veterinarians in private mixed practices and those working in regional, rural and remote locations, respectively. When years since graduation were added to the final multivariable model, there was limited evidence of confounding: regression estimates changed 17.1% for gender, 3.8% and 2.6% for workplace and 3.6% and 8.3% for location. The fit of this final model was good (Chi squared = 40.7, df = 5, *p* < 0.001).

Predictors for the administration of a premedication or sedation during an emergency euthanasia are described in Table 5. Gender, workplace, location and years since graduation were assessed in a univariable logistic regression. The type of workplace was significantly associated with the administration of a premedication or sedation in an emergency euthanasia (*p* = 0.001). Veterinarians who worked in “other” practice types were 3.2 times more likely (95% CI 1.5–6.6) to administer a premedication or sedation prior to euthanasia compared to veterinarians who worked in private companion animal practices. Gender, location and years since graduation were not significantly associated with the likelihood of administering a premedication or sedation during an emergency euthanasia. Adding “Years since graduation” to the model with workplace changed the regression estimates by 10.7% and 0.9%; therefore, the variable was not included in the final model.

### 3.4. Free Text Responses

In total, 238 respondents provided free-text responses, comprising 4805 words. Coding frequencies and examples are provided in Table 6. The most frequent codes were “pre-medication, sedation and/or analgesia” and “use of intravenous catheters”. Opinions and experiences differed widely, with some respondents strongly supportive of these measures and others firmly against. For example, some respondents felt that premedication was mandatory and helped reduce adverse effects, while others felt that it should be avoided and may increase the risk of adverse effects. Similarly, some respondents indicated that they always or mostly place an IV catheter to facilitate venous access, while others stated they would never do so, particularly as it would involve having a nurse or technician (rather than the owner) restrain the dog and may require transient separation of the owner and the dog. Overall, views about aspects of performing euthanasia, including the impacts on dogs, clients and veterinary team members, varied widely.

## 4. Discussion

This is the first study to document the euthanasia practices utilised by Australian veterinarians when performing non-emergency and emergency euthanasia in canine patients.

Almost all respondents (n = 651, 99.7%) used pentobarbitone sodium as their primary euthanasia method, administered intravenously (n = 649, 99.5%). This aligns with the findings of a survey of New Zealand veterinarians (n = 361), which reported that 99% of veterinarians used pentobarbitone as their preferred euthanasia drug for dogs [20]. Barbiturates such as pentobarbitone sodium, initially developed as anaesthetic agents, have long been considered a peaceful method of bringing about death in animals [30]. A recent literature review found that sodium pentobarbital is likely the most used barbiturate acid derivative for euthanasia of animals, with the most common route of administration being intravenous [2]. As stated by Meyer Jones in 1954,

*“the curious or concerned client may see his beloved pet put into a deep sleep from which it does not recover. As long as the intravenous injection is smooth and rapid, the client can find nothing objectionable in this method.”*

[31]

However, smooth and rapid intravenous injection may not be possible due to the condition or demeanour of the patient, lack of assistance and clinician factors including confidence and skill [23]. Intravenous injection requires restraint, which may be associated with patient fear, anxiety or distress. Dogs may experience pain associated with physical restraint, IV catheter placement, injection or extravasation of the drug [32]. Indeed, the most common justifications for using a premedication or sedation in both non-emergency and emergency situations were to reduce stress of the patient, and/or the owner, as a means of chemical restraint, respectively. For these reasons, the administration of a premedication or sedation is considered best practice, particularly for its association with reduced adverse outcomes and animal distress compared to euthanasia with pentobarbitone sodium alone [1,21,33]. In addition to reducing patient fear, anxiety and distress, effective premedication or sedation may increase practitioner confidence in their ability to access a vein for IV catheter placement or drug administration, reduce or eliminate the need for restraint, reduce the risk of adverse effects such as agonal gasping and minimise distress of both clients and veterinary team members [34]. In the context of supply chain disruption limiting access to pentobarbital in the United States and Canada in 2021, pre-euthanasia anaesthesia was recommended to eliminate pain, distress and adverse effects associated with administration of pentobarbital alternatives, including T-61, potassium chloride and magnesium sulphate [35]. While the shortage did not impact veterinarians in Australia, the experience of our North American counterparts underscores the need for familiarity with both pre-euthanasia anaesthesia and alternative euthanasia drugs.

In the current study, not all veterinarians had utilised a premedication or sedation: 33.3% of veterinarians did not use a premedication or sedation for non-emergency euthanasia. A survey of New Zealand veterinarians found that 33% of veterinarians always sedated dogs prior to euthanasia, while 20% reported they would not use sedation [20]. A smaller proportion of respondents administered a premedication or sedation in an emergency (46.4%) compared with those who did not (53.6%). Our findings align with the New Zealand study, where veterinarians were less likely to use sedation for emergency cases [20]. In emergency situations, patients can present as critical or moribund so that sedative drugs could precipitate decompensation or death in these patients. Alternatively, emergency patients may have an IV catheter placed during triage and stabilisation, facilitating venous access without the need for drugs. Patients presented for emergency care may be in acute pain and distress and, thus, already be treated with drugs that would otherwise be used for premedication or sedation prior to euthanasia. Alternatively, some patients may already be anaesthetised when the decision to euthanise is made.

The free-text responses suggest that some veterinarians avoided premedication or sedation due to concerns about adverse effects, previous negative experiences, a perceived lack of benefit and prolonging the overall euthanasia experience for the client. The potential negative impacts of premedication or sedation include increased expense; altered body physiology, rendering some techniques more difficult; a potentially unpredictable transition to sedation and potential for adverse effects (such as vomiting or dyspnoea) [34]. New Zealand veterinarians who did not usually use sedation reported that it was unnecessary, made veins difficult to find, was time-consuming to perform, may cause owners distress, it was against their clinic policy to sedate animals or that they found euthanasia smoother without sedation [20]. Robertson argues that pre-euthanasia sedation improves the experience for the animal, owner and veterinary team members and that its benefits greatly outweigh potential undesirable effects [36]. It is possible that further training in euthanasia and the availability of euthanasia protocols encompassing techniques for premedication or sedation may alleviate concerns about undesirable effects and increase the use of premedication or sedation prior to euthanasia. Indeed, the majority of New Zealand veterinarians (83%) reported having changed their euthanasia technique since graduation, with the most common change being adoption of pre-euthanasia sedation [20]. As 74% of respondents had received no additional formal training in euthanasia since graduation, these changes were likely due to experience or the existence of a clinic protocol. Of the 41% in New Zealand who worked in a clinic with a protocol for canine euthanasia, 97% of veterinarians indicated that they would follow this.

The most commonly used drugs for premedication or sedation prior to both non-emergency and emergency euthanasia were tiletamine zolazepam, acepromazine and opioids. Tiletamine-zolazepam was the most frequently used drug in non-emergency euthanasia (n = 200, 45.2%) and the second most frequently used drug in emergency euthanasia (n = 94, 31.8%). Tiletamine is a dissociative anaesthetic while zolazepam is a benzodiazepine, which, in combination, provide mild to moderate analgesia, muscle relaxation and chemical restraint in dogs, with effects occurring within minutes after intramuscular injection [37], allowing easy intravenous access to patients for the administration of their chosen euthanasia agent. This was one of the most commonly used agents prior to euthanasia in dogs in New Zealand, with 15.5% of veterinarians using tiletamine-zolazepam alone or in combination with acepromazine for pre-euthanasia sedation [20].

The most commonly used drug for premedication or sedation in emergency euthanasia, and the third most commonly used drug for non-emergency euthanasia, was opioids. These were administered by more than half (n = 147, 51.4%) of respondents in their most recent emergency euthanasia, compared to just over one third for non-emergency euthanasia (n = 154, 34.8%). The potential reasons for more frequent use in emergency euthanasia may be due to the patient’s condition, including their level of pain. Opioids are effective sedatives and are recommended as the drugs of first choice for analgesia of veterinary patients, often administered to animals with mild and severe pain [38]. Opioids were commonly combined with other agents in sedation protocols utilised by New Zealand veterinarians [20].

The second most commonly administered drug for premedication or sedation in non-emergency euthanasia, and the third most common for emergency euthanasia, was acepromazine. Acepromazine is a phenothiazine neuroleptic agent commonly used for tranquilisation or sedation [39]. There is some debate about whether acepromazine causes anxiolysis or only sedation. As a single agent, there is some concern that this drug may worsen aggression or increase startling of some dogs. However, it is often combined with an opioid to enhance analgesia (neuroleptanalgesia).

While approximately equal proportions of respondents administered premedication or sedation intravenously, subcutaneously and intramuscularly in non-emergency euthanasia, the majority of veterinarians utilised the intravenous route for emergency euthanasia. As outlined above, IV access may already be established in these patients, facilitating painless administration and more rapid onset of action from the drugs.

The differences in approaches to premedication or sedation may be due to veterinarian preference, previous euthanasia experiences, inventory, drug scheduling, onset of action, cost and the variable teaching regarding the use of premedication or sedation taught in Australasian veterinary schools [16,19]. Littlewood and colleagues reported that Australasian veterinary schools taught euthanasia by an IV barbiturate overdose, either with or without premedication or sedation [16]. This survey described a similar association, with less than 10% (non-emergency: n = 41, 9.3%, emergency: n = 22, 7.7%) of respondents reporting that they used a premedication or sedation because they were taught to. It is possible that the number of veterinarians administering premedication or sedation prior to euthanasia may increase with increased emphasis on explicit teaching of euthanasia protocols during their veterinary training. Cooney and colleagues have called for the expansion of euthanasia education in veterinary schools in the United States [19], while Littlewood and colleagues have called for explicit assessment of euthanasia competency in Australasian veterinary schools [16].

The gap between current recommendations to premedicate or sedate animals prior to euthanasia and clinical practice suggests that further training can improve veterinarians’ confidence and familiarity with pre-euthanasia medication or sedation, including appropriate doses and routes of administration to maximise benefits and minimise potential adverse effects. Veterinarians may elect to undertake continuing professional development (CPD) on euthanasia techniques. For example, the Companion Animal Euthanasia Training Academy (CAETA https://caetainternational.com/, accessed on 27 April 2023) is one organisation providing training specifically on euthanasia and end-of-life care in companion animals.

Most veterinarians euthanised dogs in the veterinary clinic or hospital, with a slightly higher proportion of emergency euthanasia (89.9% compared to 84.7% for non-emergency euthanasia) occurring in veterinary settings. This was expected, as companion animal veterinarians are less equipped to deal with emergencies when off-site. The slightly higher proportion of veterinarians euthanising dogs off site for non-emergency euthanasia may reflect the relatively recent expansion of in home euthanasia (IHE) services within Australia. Given that the majority of canine euthanasia occurs in veterinary clinical settings, it is important to ensure that a suitable environment is available within the clinic to perform euthanasia. Features of a suitable environment may include non-slip flooring, adjustable lighting and adjustable temperature [40]. Depending on patient, client and environmental factors including climate, some outdoor spaces such as gardens may be suitable. If an appropriate space is not available within the veterinary clinical setting, a house call or referral to an IHE provider may be better for the patient and client.

The majority of respondents scheduled 30 min for euthanasia appointments. Cooney and Kipperman argue that longer appointments of 45–60 min should be scheduled to facilitate discussion of prognosis, shared decision making and informed consent [32]. They argue that owners who perceived that they are “rushed through” appointments may become frustrated and upset, and that companion animal euthanasia “have evolved to be pseudo-funerals: unique, emotional medical procedures in full view of owners unlike anything else undertaken in veterinary medicine” [32]. Additionally, some discussions, including education about what to expect during euthanasia, may take place in prior appointments, over the phone or with other veterinary team members. Such discussions also give team members a chance to develop an individualised plan for the patient and ensure they are aware of the individual needs and preferences of the patient and the psychosocial needs of the owner [40]. Proactive scheduling of non-emergency euthanasia during quieter periods where possible may benefit patients, clients and even veterinary team members by providing a calm environment with minimal distractions and potentially fearful stimuli [40].

When it came to performing euthanasia, the majority of veterinarians were assisted by veterinary nurses in both non-emergency and emergency situations. This underscores the importance of training and supporting veterinary nurses to participate in euthanasia, including appropriate restraint of patients. Around one third of veterinarians were not assisted in performing their most recent euthanasia. It is possible that, in these cases, assistance was not required due to the temperament or condition of the patient. However, it may be that assistance was not available (for example, due to staff shortages). This possibility highlights the need for euthanasia protocols that can be employed by a single operator. The use of premedication or sedation may reduce the need for assistance through chemical restraint. A very small proportion of veterinarians were assisted by clients. Client assistance may be required if no other source of assistance is available. However, it is possible that some clients preferred to assist. Veterinarians involving clients in euthanasia (for example in holding or restraining animals) need to consider the safety of clients and patients.

Many respondents took adjunctive or nonpharmacological measures to improve the euthanasia experience for patients and clients; ensuring euthanasia was performed away from other animals, providing soft bedding, extending the appointment time and feeding treats were the most common. These measures are increasingly adopted by veterinary teams seeking to minimise fear, anxiety and stress in veterinary patients in general [41]. While there is little evidence supporting the efficacy of nonpharmacological interventions in minimising fear, anxiety and distress on veterinary patients, these measures have the potential to improve the comfort and experience of patients, clients and veterinary team members. They are likely most effective when tailored to individual patients, taking into account their preferences where possible.

The free-text responses revealed divergent views on a number of aspects of euthanasia, particularly premedication or sedation (as discussed), as well as the placement of intravenous catheters. This finding was similar to that of a New Zealand study, which reported a high variation of views around the placement of intravenous catheters, methods of patient restraint and the presence of owners during euthanasia [20]. Intravenous catheters reduce the risk of extravasation of medication and allow the placement of an extension set so that euthanasia drugs can be administered with some distance from the patient. However, they require a degree of restraint and usually an assistant to place and, in some cases, involved separating the dog from the owner. Current best practice suggests that, where possible, the bonded dog–owner pair should be kept together throughout the euthanasia appointment [42]. This recommendation is supported by a randomised crossover trial, which found that dogs displayed signs of increased fear, anxiety and stress when examined in the common treatment area without their owners present, compared to the examination room with their owners present [43].

Three of the four potential predictors we explored had significant associations with the administration of a premedication or sedation prior to a non-emergency euthanasia. Gender, primary workplace and location were significantly associated with the administration of a premedication or sedation in a non-emergency euthanasia. Females were 1.9 times more likely to administer a premedication or sedation compared to males. In previous studies, female veterinarians tended to estimate pain as more severe and treat animals for pain more frequently than their male counterparts [44,45,46]. It was speculated that this is because female veterinarians are more empathic [47]. Veterinarians whose primary workplace was a private mixed animal practice were least likely to administer a premedication or sedation compared to those in private companion animal practices and “other” workplaces. Those working in “other” workplaces were 3.5 times more likely to administer a premedication or sedation prior to euthanasia compared to those in private companion animal practices. As this cohort contained veterinarians identifying as mobile veterinarians, who may provide IHE services, as well as animal welfare veterinarians and those working in academia, it is possible that this cohort are more likely to be exposed to the latest euthanasia guidelines. Alternatively, for those in “other” workplaces who did not routinely work with dogs, the use of premedication or sedation may reflect varying confidence levels regarding euthanasia technique. Further studies with larger sample sizes are required to clarify this finding. Veterinarians who practice in rural and remote locations were less likely to administer a premedication or sedative compared to those in metropolitan locations. It is possible that clients in rural and remote locations may have increased practical or financial constraints, or veterinary team members may have a lack of time due to a higher workload and workforce shortages [48].

Only one predictor was significantly associated with administration of a premedication or sedation prior to emergency euthanasia. Veterinarians in the “other” variable in primary workplace were 3.2 times more likely to give a premedication or sedation compared to veterinarians in private companion animal practice. This could be because veterinarians identified in this category worked as mobile veterinarians who may provide a house-call euthanasia service or in emergency and critical care settings where patients were treated with sedation or analgesia during the initial case triage or management. As some of these veterinarians identified as working primarily with non-canine species, it may also reflect variation in confidence levels regarding euthanasia technique between different veterinary clinical settings [25]. As this is a heterogenous cohort, it is difficult to interpret this finding, and further studies with larger sample sizes would help elucidate this finding.

When analysing both non-emergency and emergency euthanasia, “year since graduation” was not associated with an increased likelihood for the administration of a premedication or sedation. This was an unexpected finding as the more recent guidelines on euthanasia of companion animals recommend pre-euthanasia premedication or sedation, and it has recently been referred to as best practice [1,19,49,50]. Therefore, we anticipated that more recent graduates (those with fewer years of experience) would be more likely to be exposed to recent guidelines and, therefore, more likely to administer premedication or sedation compared to earlier graduates (with greater years of experience).

In order to promote the use of premedication or sedation, the factors underlying these differences require further exploration. For example, if veterinarians working in mixed practice, or those working in rural and remote locations, are less likely to administer a premedication or sedation due to practical constraints such as lack of time or staff shortages, providing further training or protocols without addressing such constraints may not lead to the increased adoption of this practice. Alternatively, if some veterinarians are less likely to administer a premedication or sedation due to lack of access to training, the opportunity to undertake appropriate training is likely to be beneficial.

## 5. Limitations

Responses may be influenced by recall bias if participants did not recently perform the euthanasia [51]. We sought to limit recall bias by limiting respondents to sharing their most recent euthanasia experiences within the previous 12 months. However, how veterinarians approach their most recent cases may not reflect how they perform euthanasia in general, and therefore, the survey could not explore individual variation in euthanasia technique. One approach that may facilitate comparison is asking respondents to describe their approach to the euthanasia of a particular animal (for example, a dog of a particular breed, size, signalment and temperament), an approach taken in a contemporaneous survey [20].

Voluntary surveys such as this are subject to non-response bias, whereby they can only reflect the views and experiences of those motivated to respond to the survey [51]. Offering an incentive may have increased response rates and encouraged non-respondents to participate. Surveys may also be associated with social-desirability bias [51]. For example, respondents may have reported what they feel the investigators think should be done rather than report on actual practice. The survey was anonymous to limit the impact of social desirability bias on responses.

We did not include a definition of what constitutes a non-emergency and emergency euthanasia. Although this was not raised during piloting of the survey with veterinarians of varying experience, we acknowledge participants may have interpreted what constitutes emergency and non-emergency euthanasia differently, which may have reduced the precision with which we could determine the nature and extent of techniques used in non-emergency and emergency euthanasia.

To reduce survey attrition, we sought to reduce the length of the survey. In future, it would be useful to collect data on the frequency of canine euthanasia performed by Australian veterinarians. The mean number of dogs euthanised by New Zealand veterinarians each month was 7.2 [20]. The frequency of euthanasia performed may influence the technique utilised. Furthermore, it would be helpful to ask respondents if their clinic had a standard protocol for euthanising dogs and whether this was followed. This would aid in refining and standardising protocols. Additionally, it would be useful to collect data on dose rates and the depth and quality of sedation or anaesthesia achieved in patients given pre-euthanasia medication. It is recommended that the administration of pentobarbitone sodium via intra-organ routes (intracardiac, intrarenal and intrahepatic) only be performed in anaesthetised animals as injection into these sites is painful [23,52]. Additionally, we could collect data on the rate of drug administration. Slower rates of administration of pentobarbitone sodium in heavily sedated or anaesthetised dogs may reduce adverse effects such as agonal gasping [23]. To refine and standardise euthanasia protocols, it would be helpful to collect data on dose rates, as well as qualitative data to explore indications and contraindications for the administration of a premedication or sedation prior to euthanasia.

In the current study, we did not collect data on the quantity or quality of training in euthanasia techniques undertaken by veterinary students or graduates, which may have highlighted gaps. New Zealand veterinarians ranked their euthanasia training received in veterinary school as below satisfactory, and 74% received no formal training in euthanasia after graduation [20]. Most learned from experience or discussions with colleagues. The anonymity of the survey meant that the authors were unable to seek additional information and clarification regarding individual responses.

There were 13,933 veterinarians registered in Australia in 2021, and this survey had a sample size of 695, representing approximately 5% of the Australian veterinarian population [53]. Thus, caution must be exercised when generalising the results. Nonetheless, our sample aligned with the Australian Veterinarian Association’s 2021 Workforce survey analysis (n = 3749), with respondents to both surveys featuring a majority of female practitioners and those working with companion animals [53].

Finally, it is important to note that the time period about which respondents were surveyed overlapped at times with COVID-19-associated public health orders, including physical distancing requirements in which euthanasia practices may have been altered. For example, due to the public health orders requiring physical distancing, low and no contact euthanasia practices were utilised in many practices to minimise the risk of SARS-CoV-2 transmission between clients and veterinarians [54]. It may be, for example, that premedication or sedation were administered to facilitate placement of an IV catheter and long extension set so that a veterinarian could administer sodium pentobarbitone IV while physically distancing from the client as they held the animal in their final moments. Thus the proportion of Australian veterinarians administering premedication or sedation may be overestimated in this study.

## 6. Conclusions

This study found that the majority of veterinarians euthanised canine patients using IV pentobarbitone sodium, with just over two thirds administering premedication or sedation prior to euthanasia in non-emergency euthanasia and less than half administering premedication or sedation in emergency euthanasia. There is scope for further training in euthanasia techniques to ensure that Australian veterinarians are comfortable with best euthanasia practices, including the use of premedication or sedation, which may improve patient welfare. There is a need to ensure that veterinarians have access to appropriate training and continuing professional development around technical aspects of canine euthanasia. The development of euthanasia protocols may assist in maximising the benefits and minimising adverse effects associated with both euthanasia and premedication and sedation. The refinement of euthanasia practices may also require addressing financial and practical constraints. Our findings allow individual veterinarians to benchmark their own practices, both pharmacological and non-pharmacological, or adjunctive measures to improve the euthanasia experience for canine patients, their owners and veterinary team members involved against their peers. Additionally, it may assist in the development and refinement of euthanasia protocols.

## Figures and Tables

**Table 1:** Frequency table describing demographic information of respondents to a survey of Australian veterinarians euthanasia techniques used in dogs (n = 598).

Demographic Parameter	Category	Number	Percentage (%)
Gender (n = 598)	Male	105	17.6
	Female	490	81.9
	Other	3	0.5
Primary Workplace (n = 598)	Animal Shelter practice/charity/NGO	16	2.7
	Private Companion Animal Practice	433	72.4
	Private mixed practice	107	17.9
	Other ^+^ (please specify)	42	7.0
Location (n = 598)	Metropolitan (Major capital cities)	287	48.0
	Regional (All of the towns, small cities and areas that lie beyond capital cities)	263	44.0
	Rural (Open country and settlements fewer than 2500 residents) and Remote (places located a considerable distance from regional and metropolitan areas)	48	8.0

^+^ Other category includes veterinarians from animal shelter practice/charity/NGO, research laboratory, veterinary teaching hospital and other. NGO = non-government organisation.

**Table 2:** Frequency table describing practices relating to euthanasia of dogs in a survey of Australian veterinarians (non-emergency n = 562; emergency n = 286).

		Non-Emergency		Emergency	
		Count (n = 562)	%	Count (n = 286)	%
Location of euthanasia	House call	97	14.9	27	9.4
	At the clinic	552	84.7	257	89.9
	Other	3	0.5	2	0.7
Owner present?	Yes	622	95.4	257	89.9
	No	30	4.6	29	10.1
Length of euthanasia consult	10 min	18	2.8	-	-
	20 min	116	17.8	-	-
	30 min	386	59.2	-	-
	40 min	31	4.8	-	-
	60 min	26	4.0	-	-
	Other	60	9.2	-	-
	Unlimited	15	2.3	-	-
Were you assisted?	Yes	449	68.9	197	68.9
	No	203	31.1	89	31.1
Who assisted?	Client	8	1.8	3	1.5
	Veterinary nurse	436	97.3	192	97.5
	Other	4	0.9	2	1.0
Adjunctive measures	Away from other animals	510	78.2	202	70.6
	Pheromones	102	15.6	38	13.3
	Dim lighting	148	22.7	61	21.3
	Longer appointment time	437	67.0	126	44.1
	Soft bedding	513	78.7	175	61.2
	Soft music playing	17	2.6	5	1.7
	Treats	390	59.8	58	20.3
	Other (please specify)	89	13.7	22	7.7
	None	20	3.1	37	12.9

Please note that respondents could select multiple options.

**Table 3:** Descriptive results and univariable logistic regression results for demographic variables associated with the administration of premedication in the most recent and non-emergency euthanasia of Australian veterinarians (n = 595).

		Premeds			Univariate	
Predictor	Category	Yes (%)	No (%)	Total	OR (95% CI)	*p*-Value
Gender	Male *	58 (55.2)	47 (44.8)	105	1.0	0.004
	Female	342 (69.8)	148 (30.2)	490	1.9 (1.2–2.9)	
Workplace	Private companion animal practice *	294 (68.4)	136 (31.6)	430	1.0	<0.001
	Private mixed practice	55 (51.4)	52 (48.6)	107	0.5 (0.3–0.8)	
	Other ^+^	51 (87.9)	7 (12.1)	58	3.4 (1.5–7.6)	
Location	Rural and remote *	23 (47.9)	25 (52.1)	48	1.0	<0.001
	Metropolitan	213 (75.0)	71 (25.0)	284	3.3 (1.7–6.1)	
	Regional	164 (62.4)	99 (37.6)	263	1.8 (1.0–3.3)	
Years since graduation	0–5 years *	126 (68.8)	57 (31.2)	183	1.0	0.641
	6–10 years	65 (62.5)	39 (37.5)	104	0.7 (0.4–1.2)	
	11–25 years	117 (66.5)	59 (33.5)	176	0.9 (0.6–1.4)	
	26+ years	92 (69.7)	40 (30.3)	132	1.0 (0.6–1.7)	

^+^ Other category includes veterinarians from animal shelter practice/charity/NGO, research laboratory, veterinary teaching hospital and other. NGO = non-government organisation. * Reference category.

**Table 4:** Final binary multivariable logistic regression results for demographic variables associated with the administration of premedication in the most recent non-emergency euthanasia performed by Australian veterinarians (n = 595).

Predictor	Categories	Adjusted OR	95% CI	*p*-Value
Gender	Male *	1.0	-	0.004
	Female	1.9	1.2–3.0	
Workplace	Private companion animal practice *	1.0	-	0.003
	Private mixed practice	0.7	0.4–1.2	
	Other ^+^	3.5	1.5–7.9	
Location	Rural and Remote *	1.0	-	0.018
	Metropolitan	2.5	1.2–5.1	
	Regional	1.6	0.8–3.1	

^+^ Other category includes veterinarians from animal shelter practice/charity/NGO, research laboratory, veterinary teaching hospital and other. NGO = non-government organisation. * Reference category.

**Table 5:** Descriptive results and univariable logistic regression results for demographic variables associated with the administration of premedication in the most recent emergency euthanasia performed by Australian veterinarians (n = 515).

		Premeds			Univariate	
Predictor	Category	Yes (%)	No (%)	Total	OR (95% CI)	*p*-Value
Gender	Male *	43 (47.3)	48 (52.7)	91	1.0	0.226
	Female	230 (54.2)	194 (45.8)	424	1.3 (0.8–2.1)	
Workplace	Private companion animal practice *	196 (52.5)	177 (47.5)	373	1.0	0.001
	Private mixed practice	42 (43.3)	55 (56.7)	97	0.7 (0.4–1.1)	
	Other ^+^	35 (77.8)	10 (22.2)	45	3.2 (1.5–6.6)	
Location	Rural and remote *	23 (53.5)	20 (46.5)	43	1.0	0.422
	Metropolitan	130 (56.0)	102 (44.0)	232	1.1 (0.6–2.1)	
	Regional	120 (50.0)	120 (50.0)	240	0.9 (0.5–1.7)	
Years since graduation	0–5 years *	89 (55.9)	70 (44.1)	159	1.0	0.844
	6–10 years	49 (52.1)	45 (47.9)	94	0.8 (0.5–1.4)	
	11–25 years	74 (51.4)	70 (48.6)	144	0.8 (0.5–1.3)	
	26+ years	61 (51.7)	57 (48.3)	118	0.8 (0.5–1.3)	

^+^ Other category includes veterinarians from animal shelter practice/charity/NGO, research laboratory, veterinary teaching hospital and other. NGO = non-government organisation. * Reference category.

**Table 6:** Codes, frequencies and examples of free-text comments in response to the question “is there anything else you wish to add?” regarding euthanasia techniques used by Australian veterinarians (n = 238).

Code	Number of Comments Coded	Examples (Respondent Number)
Premedication, sedation and/or analgesia	68	“I personally think that unless the animal is stressed that sedation is not beneficial, Lethabarb [pentobarbitone sodium injection] gives a smooth euthanasia anyway. We don’t sedate every animal that needs an IV catheter in practice so I don’t quite understand vets who think all euthanasias need sedation.” (48)
		“Calm, quiet, sedated with the family present for the entire process. Peace and calm. As I would want for my own pet.” (127)
		“I find mild sedation makes the euthanasia more difficult.” (194)
		“I find that from a client perspective, moving from awake to sedated to deceased is a gentler transition than just alive then dead.” (323)
		“Depends on client request for sedation prior, temperament and anxiety of the dog, haemodynamic status.” (382)
		“Premedication should be considered mandatory.” (490)
Use of intravenous catheters	60	“I tend to take animal away for catheter placement and then return to the room just in case that part is tricky and upsetting for dog or owner.” (30)
		“I’ve used longer IV extension sets for social distancing but also to give clients personal space.” (44)
		“IV catheter placement is essential.” (113)
		“I feel very strongly that placing an IV catheter prior to euthanasia is stressful for the pet and is solely for the client and vet and not the pet’s welfare. Which is why I use IM sedation.” (207)
Communicating with clients	23	“I try to make it as stress free for all involved and discuss what I am doing before and during the event. I allow time before for questions and after for grieving. I acknowledge their relationship to their pet.” (21)
		“Listen to what the clients want and work with their pace. Watch their body language and ask if unsure.” (369)
		“Good communication with the client is a big factor in a smooth procedure.” (425)
Minimising patient fear anxiety or distress	20	“If the owner elects not to be present, I always aim to minimise the time between the owner leaving and the euthanasia.” (31)
		“If the dog is eating, I often offer them chocolate, as well as regular treats.” (126)
		“I always use calm handling methods.” (167)
Minimising owner stress	17	“sedation, when not needed, adds to the stress for the owner, who often wants to get it done and be left alone to grieve for a while, or just get out the door ASAP [as soon as possible].” (82)
		“Always check owner isn’t returning to empty house or has someone to call.” (275)
		“I try to remember this will be the last memory the owner has with the animal and act accordingly.” (337)
Adverse effects and their management or avoidance	17	“Mixing ACP [acepromazine maleate] with Zoletil [tiletamine-zolazepam] definitely takes away the sting.” (78)
		“I also always warn owners about potential agonal gasping and that the eyes will stay open and they may urinate and/or defecate themselves.” (200)
		“I always use IV catheter rather then administering the euthanasia solution off the needle as I believe it is safer and reduces the chance of extravascular injection and associated side effects.” (282)
Time of appointment	12	“I wish the clinic policy was a longer than 30 min consult time for euthanasia. So there’s enough time for the owners to grieve and for the vet to compose themselves before the next consult.” (27)
		“We try and book it at the end of a shift or when there is least clients in the building to reduce stress to client and patient.” (192)
		“cost and time efficiency are primary concerns in a shelter.” (262)
Methods of euthanasia (including route of administration)	10	“I also give the first ml of Lethabarb [pentobarbitone sodium] slowly over about 30–60 s.” (85)
		“Go slow, just like a GA induction. First 1/4 to 1/3 of expected dose, wait until animal relaxes then administer remainder. Usually very smooth with little to no agonal breathing.” (466)
Approaches to euthanasia are unique or dependent on individual patient	10	“Every case and client is unique and needs to be treated as such.” (101)
		“Wherever possible I try to meet owner and pet beforehand (usually at quality of life assessment) and the owner and I work out a mutually acceptable plan for the euthanasia.” (135)
		“It is very dog and owner dependent.” (295)
Euthanasia of anxious or aggressive animals	9	“Aggressive dogs will get heavy sedation at clinic. Sometimes sedation given at home before getting to clinic.” (75)
		“Protocol regarding premedication prior to euthanasia in our clinic is based on temperament of the dog. Aggressive/anxious animals receive either a Zoletil/ACP [acepromazine maleate] combination or Trazadone/gabapentin administered at home.” (263)
		“If aggressive or distressed when handling very different protocol. Recent aggressive dog euth had trazodone gabapentin and ACP [acepromazine maleate] at home prior to a Domitor [medetomidine hydrochloride] butorphanol IM injection at clinic.” (467)
Do not separate animal from owner at any point in the process	8	“Unless the owner doesn’t wish to be present, I will always strive to keep the animal with the owner through the entire process.” (138)
		“…routinely sedate and don’t separate the animal from owner for catheter placement.” (302)
		“Never take dog away from owner if possible.” (441)
Indications and justifications for euthanasia	8	“Clear understanding of why I perform the euthanasia in regards to the well-being of the dog eg suffering pain quality of life.” (6)
		“the patients comfort is paramount, euthanasia means a good death, not quick or at the owners convenience.” (37)
		“Discussion of options with owner to ensure they feel 110% sure of their choice and once established supporting the owner and reassuring them that they have made the correct decision (this does not apply for euthanasia of healthy animals).“ (552)
Discussing aftercare of the body costs and paying accounts	7	“We try to complete consent forms, payment etc. prior to the procedure if possible.” (139)
		“Having had a conversation about the process and the owner’s wishes for the patient afterwards (e.g., cremation) prior to helpful so they are not making decisions under stress.” (177)
Assistance during euthanasia	5	“With the nurse’s help, and ideally not with owner watching, I place an IV catheter with an extension set. Then invite owner into the consult room if they wish to be present.” (85)
		“IV catheters are sometimes used to either free up the nurse OR if the sedation didn’t kick in well enough and I was concerned that I wouldn’t have great vein access.“ (183)
Location of euthanasia	5	“Although I do most euthanasias in the clinic, I actually think in many cases it is good for them to be at home but this really isn’t practical unless you’re set up to do it.” (8)
		“Extra measures are mostly for the owner’s sake (longer consult, separate room, payment before procedure, etc) rather than for the patient.” (84)
Need to be compassionate in general	3	“Gentle hands & kind words.” (15)
		“[Empathy] and compassionate response are also very important.” (439)
Sympathy cards and memorials	3	“We take paw prints after to send to client.” (23)
		“In our clinic we offer gold paw prints and hair samples and send sympathy cards. We send flowers to significant pets (long association with the clinic). I have also recently started to take photos before and after the event with the clients permission and have printed these out and given to the client. I have found this very well received.” (65)
Minimising stress to veterinary team members including self	3	“I take pride in letting them pass smoothly. I really beat myself up if they have any side effects.” (368)
		“It’s a team effort, no vet is ever euthanasing a patient without nurse and emotional support.” (532)
Euthanasia of unowned or stray animals	1	“Unrestrained/wild dog–shoot.” (22)

Key: IV = intravenous. IM = intramuscular. GA = general anaesthetic.

## Data Availability

The data are unavailable due to ethical restrictions.

## Supplementary Materials

The following supporting information can be downloaded at: https://www.mdpi.com/article/10.3390/vetsci10050317/s1, Table S1: Survey questions—Euthanasia of dogs by Australian veterinarians: a survey of current practices; Table S2: Frequency table describing the counts and percentages of respondents answering each survey question for their most recent non-emergency euthanasia in the dog; Table S3: Frequency table describing the counts and percentages of respondents answering each survey question for their most recent emergency euthanasia in the dog.
