# Supplementary material for: Euthanasia of Dogs by Australian Veterinarians: A Survey of Current Practices
Source: Vet Sci. 2023 Apr 27;10(5):317. doi: 10.3390/vetsci10050317 (PMC10224218; doi:10.3390/vetsci10050317)
Supplement: Supplementary file 1 [file vetsci-10-00317-s001.zip › vetsci-2348250-supplementary.pdf]

veterinary sciences

MDPI

Supplementary Information

Table S1. Survey Questions - Euthanasia of dogs by Australian veterinarians: a survey of current practices.

Questions	Options
I have read the Participant Information Statement, am over the age of 18 and consent to participate.	Yes<br>No
I am a registered veterinarian in Australia.	Yes<br>No
Section 1: Dogs	
Have you euthanised a dog in the last 12 months?	Yes<br>No
Non-Emergency Euthanasia	
For the most recent, non-emergency euthanasia you performed in a dog, did you use premedication or sedation?	Yes<br>No
What was the reasoning behind using a premedication?	Chemical restraint<br>Clinic's protocols<br>Reduce stress to the owner<br>Reduce stress to the patient<br>Taught to administer a premedication prior to euthanasia drugs<br>Other (please specify)<br>(Please select all that apply.)
Please specify your answer.	(Free text response)
What was the drug used for premedication or sedation prior to the euthanasia you performed most recently	Acepromazine<br>Alfaxalone<br>Inhalation anaesthesia<br>Ketamine<br>Medetomidine / Dexmedetomidine<br>Opioids (Methadone, buprenorphine, tramadol, butorphanol, pethidine)<br>Propofol<br>Tiletamine-zolazepam<br>Thiopentone<br>Xylazine<br>Other (please specify)<br>(If you have used a combination of drugs, please select each of these.)
Please specify your answer.	(Free text response)
What was the route of administration for the premedication that you used?	Inhalation<br>Intracardiac injection<br>Intravenous injection<br>Intramuscular injection<br>Intraperitoneal injection<br>Oral administration<br>Subcutaneous injection<br>Other (please specify)<br>(Please select all that apply.)
Please specify your answer.	(Free text response)
What was the primary method that you used in your most recent non-emergency euthanasia of a dog?	Pentobarbitone sodium<br>Anaesthetic inhalation<br>Potassium chloride<br>Thiopentone<br>Other (please specify)<br>(If you have used a combination of drugs, please select each of these.)

Please specify your answer.	(Free text response)
What was the route of administration of your chosen euthanasia drug?	Intravenous injection<br>Inhalation<br>Intracardiac injection<br>Intramuscular injection<br>Intraperitoneal injection<br>Oral administration<br>Subcutaneous injection<br>Other (please specify)
Please specify your answer.	(Free text response)
Was the euthanasia a house call or did it happen at the clinic?	House call<br>At the clinic<br>Other (please specify)
Please specify your answer.	(Free text response)
Was the owner present during the euthanasia?	Yes<br>No
How long do you schedule for a routine euthanasia?	10 minutes<br>20 minutes<br>30 minutes<br>Other (please specify)
Please specify your answer.	(Free text response)
Were you assisted during the euthanasia?	Yes<br>No
Who assisted?	Client<br>Veterinary nurse<br>Other (please specify)
Please specify your answer.	(Free text response)
What adjunctive measures did you take to minimise fear/anxiety/stress in the patient?	Away from other animals<br>Pheromones<br>Dim lighting<br>Longer appointment time<br>Soft bedding<br>Soft music playing<br>Treats<br>Other (please specify)<br>None<br>(Please select all that apply.)
Please specify your answer.	(Free text response)
Did you dispense medication to the owner for the patient prior to the appointment?	Barbiturates<br>Clonidine<br>Gabapentin<br>Opioids<br>Oral acepromazine<br>Trazodone<br>Other (please specify)<br>None<br>(If you have used a combination of drugs, please select each of these.)
Please specify your answer.	(Free text response)
Emergency Euthanasia	
For the last emergency euthanasia performed in a dog in the last 12 months, did you administer a premedication?	Yes<br>No<br>Have not performed
What was the reasoning behind using a premedication?	Chemical restraint<br>Clinic's protocols<br>Reduce stress to owner<br>Reduce stress to the patient<br>Taught to administer a premedication prior to euthanasia drugs

	Other (please specify)<br>(Please select all that apply.)
Please specify your answer.	(Free text response)
What was the drug used for premedication prior to euthanasia under an emergency?	Acepromazine<br>Alfaxalone<br>Inhalation anaesthesia<br>Ketamine<br>Medetomidine / Dexmedetomidine<br>Opioids (Methadone, buprenorphine, tramadol, butorphanol, pethidine)<br>Propofol<br>Tiletamine-zolazepam<br>Thiopentone<br>Xylazine<br>Other (please specify)<br>(If you have used a combination of drugs, please select each of these.)
Please specify your answer.	(Free text response)
What was the route of administration for the premedication that you used?	Inhalation<br>Intracardiac injection<br>Intravenous injection<br>Intramuscular injection<br>Intraperitoneal injection<br>Oral administration<br>Subcutaneous injection<br>Other (please specify)<br>(Please select all that apply.)
Please specify your answer.	(Free text response)
Was the emergency euthanasia a house call or did it happen at the clinic?	House call<br>At the clinic<br>Other (please specify)
Please specify your answer.	(Free text response)
Was the owner present during the euthanasia?	Yes<br>No
Were you assisted during the euthanasia?	Yes<br>No
Who assisted?	Client<br>Veterinary nurse<br>Other (please specify)
Please specify your answer.	(Free text response)
What adjunctive measures did you take to minimise fear/anxiety/stress in the patient?	Away from other animals<br>Pheromones<br>Dim lighting<br>Longer appointment time<br>Soft bedding<br>Soft music playing<br>Treats<br>Other (please specify)<br>None<br>(Please select all that apply.)
Please specify your answer.	(Free text response)
Is there anything else you wish to add about your approach to euthanising a dog?	(Free text response)
Section 2: Cats	
Have you euthanised a cat in the last 12 months?	Yes<br>No
Non-Emergency Euthanasia	

For the most recent, non-emergency euthanasia you performed in a cat, did you use premedication or sedation?	Yes<br>No
What was the reasoning behind using a premedication?	Chemical restraint<br>Clinic's protocols<br>Reduce stress to owner<br>Taught to administer a premedication prior to euthanasia drugs<br>Other (please specify)<br>(Please select all that apply.)
Please specify your answer.	(Free text response)
What was the drug used for premedication or sedation prior to the euthanasia you performed most recently?	Acepromazine<br>Alfaxalone<br>Inhalation anaesthesia<br>Ketamine<br>Medetomidine / Dexmedetomidine<br>Opioids (Methadone, buprenorphine, tramadol, butorphanol, pethidine)<br>Propofol<br>Tiletamine-zolazepam<br>Thiopentone<br>Xylazine<br>Other (please specify)<br>(If you have used a combination of drugs, please select each of these.)
Please specify your answer.	(Free text response)
What was the route of administration for the premedication that you used?	Inhalation<br>Intracardiac injection<br>Intravenous injection<br>Intramuscular injection<br>Intraperitoneal injection<br>Oral administration<br>Subcutaneous injection<br>Other (please specify)<br>(Please select all that apply.)
Please specify your answer.	(Free text response)
What was the primary method that you used in your most recent non-emergency euthanasia of a cat?	Pentobarbitone sodium<br>Anaesthetic inhalation<br>Potassium chloride<br>Thiopentone<br>Other (please specify)<br>(If you have used a combination of drugs, please select each of these.)
Please specify your answer.	(Free text response)
What was the route of administration of your chosen euthanasia drug?	Intravenous injection<br>Inhalation<br>Intracardiac injection<br>Intramuscular injection<br>Intraperitoneal injection<br>Oral administration<br>Subcutaneous injection<br>Other (please specify)
Please specify your answer.	(Free text response)
Was the euthanasia a house call or did it happen at the clinic?	House call<br>At the clinic<br>Other (please specify)
Please specify your answer.	(Free text response)
Was the owner present during the euthanasia?	Yes<br>No
How long do you schedule for a routine euthanasia?	10 minutes<br>20 minutes

	30 minutes<br>Other (please specify)
Please specify your answer.	(Free text response)
Were you assisted during the euthanasia?	Yes<br>No
Who assisted?	Client<br>Veterinary nurse<br>Other (please specify)
Please specify your answer.	(Free text response)
What adjunctive measures did you take to minimise fear/anxiety/stress in the patient?	Away from other animals<br>Pheromones<br>Dim lighting<br>Longer appointment time<br>Soft bedding<br>Soft music playing<br>Treats<br>Cat only consult room<br>ISFM accreditation<br>Other (please specify)<br>None<br>(Please select all that apply)
Please specify your answer.	(Free text response)
Did you dispense medication to the owner for the patient prior to the appointment?	Barbiturates<br>Clonidine<br>Gabapentin<br>Opioids<br>Oral acepromazine<br>Trazodone<br>Other (please specify)<br>None<br>(If you have used a combination of drugs, please select each of these.)
Please specify your answer.	(Free text response)
Emergency Euthanasia	
For the last emergency euthanasia performed in a cat in the last 12 months, did you administer a premedication?	Yes<br>No<br>Have not performed
What was the reasoning behind using a premedication?	Chemical restraint<br>Clinic's protocols<br>Reduce stress to owner<br>Reduce stress to the patient<br>Taught to administer a premedication prior to euthanasia drugs<br>Other (please specify)<br>(Please select all that apply.)
Please specify your answer.	(Free text response)
What was the drug used for premedication prior to euthanasia under an emergency?	Acepromazine<br>Alfaxalone<br>Inhalation anaesthesia<br>Ketamine<br>Medetomidine / Dexmedetomidine<br>Opioids (Methadone, buprenorphine, tramadol, butorphanol, pethidine)<br>Propofol<br>Tiletamine-zolazepam<br>Thiopentone<br>Xylazine<br>Other (please specify)<br>(If you have used a combination of drugs, please

	select each of these.)
Please specify your answer.	(Free text response)
What was the route of administration for the premedication that you used?	Inhalation<br>Intracardiac injection<br>Intravenous injection<br>Intramuscular injection<br>Intraperitoneal injection<br>Oral administration<br>Subcutaneous injection<br>Other (please specify)<br>(Please select all that apply.)
Please specify your answer.	(Free text response)
Was the emergency euthanasia a house call or did it happen at the clinic?	House call<br>At the clinic<br>Other (please specify)
Please specify your answer	(Free text response)
Was the owner present during the euthanasia?	Yes<br>No
Were you assisted during the euthanasia?	Yes<br>No
Who assisted?	Client<br>Veterinary nurse<br>Other (please specify)
Please specify your answer.	(Free text response)
What adjunctive measures did you take to minimise fear/anxiety/stress in the patient?	Away from other animals<br>Pheromones<br>Dim lighting<br>Longer appointment time<br>Soft bedding<br>Soft music playing<br>Treats<br>Cat only consult room<br>ISFM accreditation<br>Other (please specify)<br>None<br>(Please select all that apply.)
Please specify your answer.	(Free text response)
Is there anything else you wish to add about your approach to euthanising a cat?	(Free text response)
Section 3: Demographics	
Gender:	Male<br>Female<br>Other
Number of years since graduation:	(Free text response) (Please enter a whole number)
Please select the most suitable option that best describes your primary workplace.	Animal Shelter practice/charity/NGO<br>Private companion animal practice<br>Private mixed practice<br>Research laboratory<br>Veterinary teaching hospital<br>Other (please specify)
Please specify your answer.	(Free text response)
Please select the most suitable option that best describes the geographical location of your workplace.	Metropolitan (Major capital cities)<br>Regional (All of the towns, small cities and areas that lie beyond the major capital cities)<br>Rural (Open country and settlements fewer than 2,500 residents)<br>Remote (Places that are considerably out of the way and excluded from civilization)

Participant email address collection survey

If you wish to receive the overall results of this study, please provide us with your email address and submit this survey.	(Free text response)

Table S2. Frequency table describing the counts and percentages of respondents answering each survey question for their most recent non-emergency euthanasia in a dog.

Non-Emergency	Category	Number	Percentage (%)
Have you euthanised a dog in the last 12 months? (n= 690)	Yes	668	96.8
	No	22	3.2
For the most recent, non-emergency euthanasia you performed in a dog, did you use premedication or sedation? (n = 653)	Yes	442	67.7
	No	211	32.3
What was the reasoning behind using a premedication? (n=442) (Participant could select multiple options)	Chemical restraint	162	36.7
	Clinic's protocols	69	15.6
	Reduce stress to owner	363	82.1
	Reduce stress to patient	410	92.8
	Taught to administer a premedication prior to euthanasia drugs	41	9.3
	Other (please specify): to reduce adverse effects such as agonal gasping (14); to reduce stress to the veterinarian (11); to facilitate patient handling (2); for safety (2); to give the owners a time to say goodbye (2); it is my choice as part of humane euthanasia (1); the patient suffers anxiety and has medications for this (1); it is nicer (1); I always do it (1); all animals deserve to be sedated before euthanasia (1).	36	8.1
What was the drug used for premedication or sedation prior to the euthanasia you performed most recently? (n=442) (Participant could select multiple options)	Acepromazine	170	38.5
	Alfaxalone	53	12.0
	Inhalation anaesthesia	1	0.2
	Ketamine	24	5.4
	Medetomidine / Dexmedetomidine	70	15.8
	Opioids (Methadone, buprenorphine, tramadol, butorphanol, pethidine)	154	34.8
	Propofol	29	6.6
	Tiletamine-zolazepam	200	45.2
	Thiopentone	12	2.7
	Xylazine	27	6.1
	Other (please specify): benzodiazepines (midazolam (4), clonazepam (1), diazepam (1)); prilocaine (1); gabapentin (1); detomidine/acepromazine/midazolam/ketamine (1).	9	2.0
What was the route of administration for the premedication that you used? (n=442) (Participant could select multiple options)	Inhalation	0	0.0
	Intracardiac injection	0	0.0
	Intravenous injection	165	37.3
	Intramuscular injection	154	34.8
	Intraperitoneal injection	1	0.2
	Oral administration	5	1.1
	Subcutaneous injection	159	36.0
	Other (please specify)	0	0.0
What was the primary method that you used in your most recent non-emergency euthanasia of a dog? (n=653)	Pentobarbitone sodium	651	99.7
	Anaesthetic inhalation	1	0.2
	Potassium chloride	2	0.3
	Thiopentone	4	0.6
	Other (please specify): injectable anaesthetic added (2).	2	0.3

(Participant could select multiple options)			
What was the route of administration of your chosen euthanasia drug?<br>(n=652)<br>(Participant could only select one option)	Intravenous injection<br>Inhalation<br>Intracardiac injection<br>Intramuscular injection<br>Intraperitoneal injection<br>Oral administration<br>Subcutaneous injection<br>Other (please specify): intrahepatic (1); intrarenal (1).	649<br>0<br>0<br>0<br>0<br>1<br>0<br>2	99.5<br>0.0<br>0.0<br>0.0<br>0.0<br>0.2<br>0.0<br>0.3
Was the euthanasia a house call or did it happen at the clinic?<br>(n=652)<br>(Participant could only select one option)	House call<br>At the clinic<br>Other (please specify): at a shelter or pound (2); both a home and clinic (1).	97<br>552<br>3	14.9<br>84.7<br>0.5
Was the owner present during the euthanasia?<br>(n=652)	Yes<br>No	622<br>30	95.4<br>4.6
How long do you schedule for a routine euthanasia?<br>(n=652)<br>(Participant could only select one option)	10 minutes<br>20 minutes<br>30 minutes<br>40 minutes<br>60 minutes<br>Unlimited<br>Other (please specify): not able to schedule (emergency or walk-in clinic) (16); 45 minutes (15); 15 minutes (8); 60 minutes (4); 30-60 minutes (3); 40-60 minutes (3); 15-30 minutes (2); 30 minutes with nurse + 15 minutes with vet (2); 10-30 minutes (1); 15-20 minutes (1); 15-35 minutes (1); 50 minutes (1); 90 minutes (1); routine daily pound euthanasia – set aside an hour a day for the list (1); at owner's discretion (1).	18<br>116<br>386<br>31<br>26<br>15<br>60	2.8<br>17.8<br>59.2<br>4.8<br>4.0<br>2.3<br>9.2
Were you assisted during the euthanasia?<br>(n=652)	Yes<br>No	449<br>203	68.9<br>31.1
Who assisted?<br>(n=448)<br>(Participant could only select one option)	Client<br>Veterinary nurse<br>Other (please specify): other veterinarian (n=2), combined client and nurse (1), shelter staff (1).	8<br>436<br>4	1.8<br>97.3<br>0.9
What adjunctive measures did you take to minimise fear/anxiety/stress in the patient?<br>(n=652)<br>(Participant could select multiple options)	Away from other animals<br>Pheromones<br>Dim lighting<br>Longer appointment time<br>Soft bedding<br>Soft music playing<br>Treats<br>None<br>Other (please specify): owner(s) present (14); owner holding the dog (11); interacting with animals gently/calmly/in a way to minimise fear/anxiety/distress (11); performing euthanasia in a quiet space or booking the appointment at a quiet time of day (7); application of local anaesthetic prior to intravenous injection or catheterisation (5); use of an intravenous extension set to facilitate owner holding the dog during euthanasia (5); using the patient's own bedding (2); location (performing in-home euthanasia (11), using a dedicated euthanasia room (4); performing euthanasia in a garden or park (4), use of essential oils or aromatherapy (2); euthanasing a dog in a car to avoid moving them (2), performing euthanasia in a dog's favourite	510<br>102<br>148<br>437<br>513<br>17<br>390<br>20<br>88	78.2<br>15.6<br>22.7<br>67.0<br>78.7<br>2.6<br>59.8<br>3.1<br>13.5

	location (1)); performing euthanasia on the floor/ground as opposed to an examination table (2); see clients immediately (2); lighting a candle (2); feeding food that might otherwise be toxic such as chocolate (1); placing a towel over the patient's head (1); sedation (1).		
Did you dispense medication to the owner for the patient prior to the appointment? (n=652)<br>(Participant could select multiple options)	Barbiturates<br>Clonidine<br>Gabapentin<br>Opioids<br>Oral acepromazine<br>Trazodone<br>None<br>Other (please specify): diazepam (1), CBD oil (1), trazadone and gabapentin (1), detomidine (1)	0<br>2<br>29<br>2<br>7<br>26<br>607<br>4	0.0<br>0.3<br>4.4<br>0.3<br>1.1<br>4.0<br>93.1<br>0.6

Table S3. Frequency table describing the counts and percentages of respondents answering each survey question for their most recent emergency euthanasia in a dog.

Emergency:	Category	Number	Percentage (%)
For the last emergency euthanasia performed in a dog in the last 12 months, did you administer a premedication? (n= 616)	Yes<br>No<br>Have not performed	286<br>247<br>83	46.4<br>40.1<br>13.5
What was the reasoning behind using a premedication? (n=286)<br>(Participant could select multiple options)	Chemical restraint<br>Clinic's protocols<br>Reduce stress to owner<br>Reduce stress to the patient<br>Taught to administer a premedication prior to euthanasia drugs<br>Other (please specify): to provide analgesia (21); to reduce stress to the veterinarian (8); to reduce adverse effects such as agonal gasping (7); for safety (1); as treatment for a condition (dog was seizuring)(1).	114<br>30<br>184<br>259<br>22<br>38	39.9<br>10.5<br>64.3<br>90.6<br>7.7<br>13.3
What was the drug used for premedication prior to euthanasia under an emergency? (n=286)<br>(Participant could select multiple options)	Acepromazine<br>Alfaxalone<br>Inhalation anaesthesia<br>Ketamine<br>Medetomidine / Dexmedetomidine<br>Opioids (Methadone, buprenorphine, tramadol, butorphanol, pethidine)<br>Propofol<br>Tiletamine-zolazepam<br>Thiopentone<br>Xylazine<br>Other (please specify): benzodiazepines (midazolam (3), diazepam (3)).	65<br>32<br>1<br>7<br>43<br>147<br>26<br>94<br>10<br>10<br>6	22.7<br>11.2<br>0.3<br>2.4<br>15.0<br>51.4<br>9.1<br>31.8<br>3.5<br>3.5<br>2.1
What was the route of administration for the premedication that you used? (n=286)<br>(Participant could select multiple options)	Inhalation<br>Intracardiac injection<br>Intravenous injection<br>Intramuscular injection<br>Intraperitoneal injection<br>Oral administration<br>Subcutaneous injection<br>Other (please specify): intranasal (1); per rectum (1).	0<br>0<br>146<br>96<br>1<br>1<br>52<br>2	0.0<br>0.0<br>51.0<br>33.6<br>0.3<br>0.3<br>18.2<br>0.7
Was the emergency euthanasia a house call or did it happen at the clinic?	House call<br>At the clinic<br>Other (please specify): dog racing track (2).	27<br>257<br>2	9.4<br>89.9<br>0.7

(n=286)<br>(Participant could only select one option)			
Was the owner present during the euthanasia?<br>(n=286)	Yes	257	89.9
	No	29	10.1
Were you assisted during the euthanasia?<br>(n=286)	Yes	197	68.9
	No	89	31.1
Who assisted?<br>(n=197)<br>(Participant could only select one option)	Client	3	1.5
	Veterinary nurse	192	97.5
	Other (please specify): staff member (1), council ranger (1).	2	1.0
What adjunctive measures did you take to minimise fear/anxiety/stress in the patient?<br>(n=286)<br>(Participant could select multiple options)	Away from other animals	202	70.6
	Pheromones	38	13.3
	Dim lighting	61	21.3
	Longer appointment time	126	44.1
	Soft bedding	175	61.2
	Soft music playing	5	1.7
	Treats	58	20.3
	None	37	12.9
	Other (please specify): owner(s) present (4); interacting with animals gently/calmly/in a way to minimise fear/anxiety/distress (4); owner holding the dog (2); use of essential oils or aromatherapy (2); performing euthanasia in a garden or park (1); sedation (1); use of an intravenous extension set to facilitate owner holding the dog during euthanasia (1); performing euthanasia on the floor/ground as opposed to an examination table (1); administering analgesia (1); location (performing euthanasia in a space where the dog was comfortable)(1); providing oxygen to a dog in respiratory distress (1); application of local anaesthetic prior to intravenous injection or catheterisation (1); taking swift and calm action (1); in a setting where appointments are not made, ensuring owners can spend as long as they feel they need (1).	22	7.7

Please note: Percentages may not add to 100.0 due to rounding.
